# The effect of alpha-2 adrenergic receptors on memory retention deficit induced by rapid eye movement sleep deprivation 

**DOI:** 10.22038/ijbms.2020.44891.10468

**Published:** 2020-12

**Authors:** Yaser Norozpour, Mohammad Nasehi, Vahid Sabouri-Khanghah, Mohammad Nami, Salar Vaseghi, Mohammad-Reza Zarrindast

**Affiliations:** 1 Department of Cognitive Neuroscience, Institute for Cognitive Science Studies (ICSS), Tehran, Iran; 2 Cognitive and Neuroscience Research Center (CNRC), Amir-Almomenin Hospital, Tehran Medical Sciences, Islamic Azad University, Tehran, Iran; 3 Department of Neuroscience, School of Advanced Medical Sciences and Technologies, Shiraz University of Medical Sciences, Shiraz, Iran; 4 Shiraz Neuroscience Research Center, Shiraz University of Medical Sciences, Shiraz, Iran; 5 Department of Pharmacology, School of Medicine, Tehran University of Medical Sciences, Tehran, Iran

**Keywords:** Clonidine, Hippocampus, Memory, Sleep, Yohimbine

## Abstract

**Objective(s):**

Evidence shows that sleep deprivation (SD) disrupts the formation of hippocampus-related memories. Moreover, α2 adrenergic receptors that are wildly expressed in the CA1 hippocampal region have a significant role in modulating both sleep and memory formation. In the present research, we wanted to investigate the effect of stimulation and blockage of CA1 α2 adrenergic receptors by clonidine (an agonist of α2 adrenergic receptor) and yohimbine (an antagonist of α2 adrenergic receptor), respectively, on memory retention impairment induced by REM SD (RSD) in rats.

**Materials and Methods:**

Multiple platform apparatus were used to induce RSD, and the passive avoidance task was used to assess memory consolidation. Clonidine and yohimbine were injected intra-CA1.

**Results:**

The results showed that RSD (for 24 and 36, but not 12 hr) decreased memory retention, with no effect on locomotion. Post-training intra-CA1 infusion of a subthreshold dose of yohimbine (0.001 μg/rat) did not alter, while clonidine (0.1 μg/rat) restored memory retention impairment induced by RSD (24 and 36 hr). Also, none of the interventions did not influence locomotor activity.

**Conclusion:**

Our data strongly showed that CA1 α2 adrenergic receptors have a critical role in RSD-induced memory retention impairment.

## Introduction

Sleep is a circadian rhythm that is involved in modulating physiological processes (1). It has a main role in modulating cognitive functions (2). Sleep consists of non-rapid eye movement (non-REM) and rapid eye movement (REM) stages (3). Cognitive functions including learning, memory, decision making, reaction time, attention, etc., are affected by sleep, and one of the most important functions of sleep is mediating memory formation (3-5). Also, sleep has a critical role in the consolidation of lately acquired memories for long-term storage (6). Thus, sleep deprivation (SD) can negatively affect hippocampus-dependent learning and memory and long-term potentiation (LTP) (7). Furthermore, previous studies have reported the role of REM sleep in learning and memory consolidation (8, 9). 

Noradrenaline has a critical role in SD-induced cognitive deficits (10, 11). It has been reported that locus coeruleus (LC) ascending noradrenergic projections which lead to the prefrontal cortex and the hippocampus play a key role in cognitive deficits induced by SD (12). It has been also revealed that noradrenaline and adrenergic receptors modulate learning and memory processes (13). Adrenergic receptors are highly expressed in the posterior hippocampus region (CA1) that mediates learning and memory (3). Noradrenaline, by two receptor categories named α and β that couple to a G protein, exerts its own effects (12). The α adrenergic receptors consist of α1 and α2 subgroups which are expressed in a large amount in the central nervous system (CNS). Most of the α1 adrenergic receptors are post-synaptic, while α2 adrenergic receptors are both post- and pre-synaptic (14). The α2-adrenoceptors consist of various subtypes: α2A, α2B, and α2C (15). The α2A-subtype is the key α2-adrenoceptor which has an important role in different responses that are induced by the activation of α2-adrenoceptor, including modulating nociception, cardiovascular function, sedation, and releasing noradrenaline (16).

It has been shown that α2-adrenoceptors are involved in increased sleepiness following SD (11), and the regulatory role of adrenergic receptors may induce a homeostatic response to the continuous discharge by hypocretin/orexin neurons that would occur during enforced wakefulness (17). Now the question is whether RSD attenuates cognitive functions and how can we prevent it? To achieve this goal, we designed this study to investigate the impairment effect of RSD on different brain regions and the role of direct activation or deactivation of various receptors in RSD-induced responses. According to these mentioned points, this study aims to explore the effect of α2 agonism and antagonism on memory deficits induced by RSD.

## Materials and Methods

***Animals***

Ninety-six 220–250 g adult male Wistar rats were used. The animals were stored for one week in the lab before performing experiments to habituate with temperature, humidity, and circadian rhythm. The temperature was kept at 23 °C. The rats had free access to food and water. Each group consisted of eight rats and each rat was used only once. All experiments were carried out during the light phase. Our experimental protocol in this study was done accord with the Ethics Guidelines of the Institute for Cognitive Science Studies.

***Stereotaxic surgery***

All rats were anesthetized by intraperitoneal (IP) injection of ketamine hydrochloride (50 mg/kg) plus xylazine (5 mg/kg). Then, each rat was placed in a stereotaxic apparatus (Stoelting Co, Illinois, USA), the skin was incised and the skull was cleaned. Next, two steel 22-gauge guide cannulae (0.7 mm diameter) were bilaterally implanted into the CA1 region according to the atlas of Paxinos and Watson (AP: -2 mm from bregma, L: ±1.6 from the sagittal suture and V: -1.5 mm from the skull surface) (18). Cannulae were secured by dental acrylic. Rats had 7 days to recover from stereotaxic surgery and the side effects of ketamine hydrochloride and xylazine (19).

***REM sleep deprivation apparatus***

To induce RSD in rats, we used a multiple platform apparatus. The rats were placed in a water tank (90×50×50 cm) that had several small transparent Plexiglas platforms with a diameter of 7 cm and a height of 10 cm. These platforms were used by rats to avoid getting wet and for sleeping. The water surrounded the platforms (2 cm lower than the platforms). The rats were placed on these platforms to avoid getting wet and to sleep. As soon as the rats went to the REM phase, their muscle tone was significantly attenuated, and they fell into the water and woke up. Within this apparatus, all rats had enough food and water (20). 

***Intra-CA1 administrations***

For the intra-CA1 administrations, all rats were gently restrained by hand and 27-gauge injection needles were placed into the guide-cannula (1 mm below the tip of the guide cannula). All infusions were manually performed at a volume of 1 µl/rat (0.5 µl in each side) within 60 sec. Then, the rats were transferred to their cages (21).

***Step-through inhibitory avoidance apparatus***

Step-through inhibitory avoidance (IA) apparatus consisted of a trough-shaped alley which had two compartments separated by a sliding door: a dark compartment where the rats were shocked (20 × 20 × 30 cm) and a light compartment (20 × 20 × 30 cm). A sliding door (7×9 cm) was in the center of the partition between the dark and the light compartments. Foot shocks were produced by stainless steel grids (2.5 mm in diameter) which were placed at 1 cm intervals on the dark compartment’s floor. An insulated stimulator transferred periodic electric shocks (50 Hz, 3 sec, 0.1 mA intensity) to the grid floor of the dark compartment.

***Behavioral procedures***

*Training*

The training was performed according to our previous research (22-24). All rats had enough time to habituate in the lab. On the habituation day, each rat was placed only in the light compartment of the apparatus and given 30 sec to explore. Five seconds later, the sliding door was raised and opened and the rat could explore freely. Rats that waited more than 100 sec to enter the dark compartment were eliminated from the tests. When each rat entered the dark compartment with all four paws, the sliding door closed. Then, the rat received an inescapable foot shock (50 Hz, 3 sec, 0.1 mA intensity) and immediately afterward was removed from the apparatus (Habituation trial). Thirty mins later, the acquisition trial was performed. On acquisition day, each rat was placed only in the light compartment and had five sec to explore. Five sec later, the sliding door was raised. As soon as the rat completely entered the dark compartment, the sliding door was closed and the latency to enter was recorded. A footshock (50 Hz, 3 sec, 0.1 mA intensity) was transferred 3 sec after closing the door. Twenty sec after the footshock each rat was removed and placed gently into its cage. After 2 mins, the method was repeated. The training was ended when the rat stayed in the light compartment for 120 sec. The number of entries into the dark compartment (trials) was recorded. Each rat learned with a maximum of three trials.

*Retrieval test*

The retrieval test was carried out 24 hr after the training. On this day, each rat was placed into the light compartment for 20 sec, while the sliding door was raised. When the rat completely entered the dark compartment, the sliding door was closed, and the latency to enter the dark compartment was recorded. Then, each rat gently returned to its cage. A cut-off time of 300 sec was considered for this test; on the retrieval test day, no shock was delivered.

*Locomotor activity evaluation*

The locomotion apparatus (BorjSanatAzma Co, Tehran, Iran) included a clear perspex container box (30 cm × 30 cm × 40 cm high). This apparatus had a gray perspex panel (30 cm × 30 cm × 2.2 cm thick) with sixteen photocells. Photocells divided the box into sixteen equal-sized squares. The number of entries of each rat from one square to another square within five mins determined the locomotor activity.

***Drugs***

Clonidine (an agonist of α2 receptor) and yohimbine (an antagonist of α2 receptor) were procured from Tocris (Tocris Bioscience United Kingdom). All drugs were dissolved in the saline solution immediately prior to administrations at the doses of 0.001, 0.01, and 0.1 µg for each rat.

***Statistical analysis***

For evaluating the effects of post-training intra-CA1 infusions of clonidine and yohimbine, we used one-way ANOVA. Also, for evaluating the interaction effect between drugs and sleep deprivation, we used two-way ANOVA. Following a significant F value, further analyses for paired-group comparisons were performed using a post-hoc Turkey’s test. In all analyses, *P*<0.05 was considered statistically significant.

***Experimental design***

*The effects of RSD on memory consolidation and locomotor activity with or without yohimbine/clonidine*

In this test, 12 groups of rats were used. All rats were treated with RSD for 0, 12, 24, and 36 hr. The rats received intra-CA1 administration of saline (saline 1 µl/rat; four groups), subthreshold dose of yohimbine (0.001 µg/rat; four groups), and subthreshold dose of clonidine (0.1 µg/rat; four groups) immediately after training. 

## Results

***The effects of RSD on memory consolidation and locomotor activity with or without yohimbine/clonidine***

**Figure 1:**
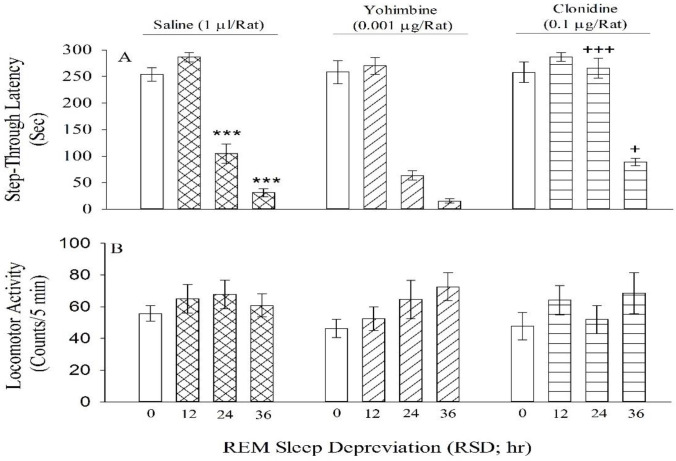
The effect of rapid eye movement sleep deprivation (RSD) on memory consolidation (panel A) and locomotion (panel B) with or without yohimbine or clonidine. Each group (saline 1 µl/rat, yohimbine 0.001 µg/rat, clonidine 0.1 µg/rat) consisted of four sub-groups which were treated with RSD at different hours (0, 12, 24, and 36). Data are expressed as Mean±SEM of eight rats in each group. ***P<0.001 in comparison with saline 1 µl/rat + 0 hr RSD. +*P*<0.05 and +++*P*< 0.001 in comparison with respective sub-groups of the saline group

One-way ANOVA and post-hoc analysis revealed that RSD for 24 and 36 but not 12 hr decreased memory consolidation [F (3, 28) = 32.21, *P*<0.001, Figure 1A], even though it did not influence locomotor activity [F (3, 28) =2.4, *P*>0.05, Figure 1B] by itself.

Two-way ANOVA showed that subthreshold dose of yohimbine (0.001 μg/rat) did not influence dose-response curve induced by RSD-treated rats on memory consolidation [treatment effect: F (1, 56) = 3.24, *P*>0.05; dose effect: F (3, 56) = 23.4, *P*<0.001; treatment and dose interaction effect: F (3, 56) =2.9, *P*>0.05, Figure 1A] and locomotor activity [treatment effect: F (1, 56) =0.661, *P*>0.05; dose effect: F (3, 56) = 4.4, *P*>0.05; treatment and dose interaction effect: F (3, 56) = 1.18, *P*> 0.05, Figure 1B].

 Two-way ANOVA also showed that subthreshold dose of clonidine (0.1 μg/rat) altered response curve induced by RSD on memory consolidation [treatment effect: F (1, 56) =8.64, *P*<0.001; dose effect: F (3, 56) =45.24, *P*<0.001; treatment and dose interaction effect: F (3, 56) =5.24, *P*<0.05, Figure 1A], while it did not alter locomotor activity [treatment effect F (1, 56) =0.572, *P*>0.05; dose effect: F (3, 56) =3.62, *P*>0.05; treatment and dose interaction effect: F (3, 56) =2.62, *P*>0.05, Figure 1B]. *Post hoc* analysis revealed that clonidine restored memory consolidation impairment induced by RSD (24 and 36 hr). 

## Discussion

In line with previous studies, the results of this research affirmed that RSD impairs memory consolidation (3, 25, 26). Sleep disturbances impair different types of learning and memory in animal models, such as passive avoidance learning (27). Therefore, from a functional point of view, RSD has negative effects on various cognitive performances, especially attention, working memory, decision making, and long-term memory (7, 8). Previous studies have reported that RSD for 24 hr disrupts memory formation in inhibitory passive avoidance task (4, 28). Another study has also revealed that SD for 72 hr leads to passive avoidance retention deficit (29). It’s important to note that, SD induces oxidative stress via an increase in lipid peroxidation and nitric oxide, and via reducing the glutathione level (29). Oxidative stress interferes with important hippocampal functional roles and induces cognitive decline, including memory deficit (30). Also, increased oxidative stress in the hippocampus may have a role in the impairment of spatial memory retention (31). The side effects of oxidative stress are more prominent in the hippocampus than in the cortex. This effect reflects the great vulnerability of this brain area in comparison with the other brain regions (32). 

We reported that intra-CA1 infusion of a subthreshold dose of yohimbine did not alter memory consolidation impairment induced by RSD. On the contrary, intra-CA1 infusion of a subthreshold dose of clonidine restored RSD-induced amnesia (24 and 36 hr). None of the previous interventions influenced locomotor activity. Our previous study has shown that both clonidine and yohimbine (only in 24 hr) restore memory retention impairment induced by total sleep deprivation (TSD) in rats. It has been revealed that α2-adrenoreceptors are associated with synaptic plasticity, learning, and memory (28). Clonidine, the α2-adrenergic agonist, has shown its enhancement effect on learning and memory in different studies (33, 34). The activation of neurons in the LC region undergoes certain rhythms that are significantly associated with the sleep/wakefulness cycle. These neurons fire at the highest rates when animals are active and awake, and fire at the lower rates when animals are in both non-REM and REM phases of sleep (8). Therefore, synaptic plasticity would be increased if synaptic activation synchronously enhanced in the hippocampus with elevating the spiking rate of LC neurons; for example, synaptic plasticity seems to occur more easily within active waking periods. However, a previous study showed that sleep is significantly involved in the consolidation of declarative memory (35) because SD potently impairs long-term synaptic plasticity in the rats’ hippocampus (36).

Noradrenaline may have a role in enhancing memory via an increase in general arousal (37, 38). It has been shown that noradrenaline has the main role in memory encoding and consolidation via pathways from the basolateral amygdala (BLA) to the entorhinal cortex, hippocampus, or anterior cingulate cortex (ACC) (39). On the other hand, noradrenaline is not only restricted to the amygdala, but also influences many brain areas including neocortical and hippocampal regions, and has interactions with other neurotransmitters, such as acetylcholine (ACh) (40, 41). It has been suggested that these effects of noradrenaline may be correlated with its ability to stabilize early LTP and transform it into late LTP (42). 

As discovered, the LC fires tonically during wakefulness and induces stimulus-related bursts during activity (37, 43). This tonic rate is related to the alertness level. During the non-REM phase, the tonic rate of LC is slowly attenuated with sleep depth. Within the REM phase, LC neurons cease firing altogether (44, 45); however, during phasic slow-wave sleep which contains sleep spindles and slow oscillations, LC neurons exhibit burst firing activity (45). The clonidine’s restoration effect on RSD-induced memory deficit is unclear and needs further investigations, but it seems that clonidine can reverse the impairment effect of both TSD and RSD on memory formation. In addition, the role of clonidine in regulating glutamatergic transmissions through stimulation of G-protein-coupled adrenergic receptors may have a role. Although further studies are needed, the present research is an important step in investigating the role of CA1 α2 adrenoreceptor activation in RSD-induced amnesia. 

## Conclusion

Our results showed that RSD for 24 and 36 hr decreased memory consolidation. It seems that oxidative stress following RSD has the main role in this impairment effect. Yohimbine did not alter memory function, but clonidine restored the impairment effect of RSD on memory formation. The firing rate of LC neurons during wakefulness, Non-REM and REM sleep, and also, modulating the efficacy of glutamatergic transmissions through activation of G-protein-coupled alpha-2 adrenergic receptors by clonidine may lead to the restoration effect of clonidine on RSD-induced memory consolidation deficit.
